# Modeling disagreement in automatic data labeling for semi-supervised learning in Clinical Natural Language Processing

**DOI:** 10.3389/frai.2024.1374162

**Published:** 2024-10-02

**Authors:** Hongshu Liu, Nabeel Seedat, Julia Ive

**Affiliations:** ^1^Department of Computing, Imperial College London, London, United Kingdom; ^2^Department of Applied Mathematics and Theoretical Physics, University of Cambridge, Cambridge, United Kingdom; ^3^School of Electronic Engineering and Computer Science, Queen Mary University of London, London, United Kingdom

**Keywords:** automated labeling, clinical text, Natural Language Processing, radiology, semi-supervised learning, uncertainty, Gaussian processes

## Abstract

**Introduction:**

Computational models providing accurate estimates of their uncertainty are crucial for risk management associated with decision-making in healthcare contexts. This is especially true since many state-of-the-art systems are trained using the data which have been labeled automatically (self-supervised mode) and tend to overfit.

**Methods:**

In this study, we investigate the quality of uncertainty estimates from a range of current state-of-the-art predictive models applied to the problem of observation detection in radiology reports. This problem remains understudied for Natural Language Processing in the healthcare domain.

**Results:**

We demonstrate that Gaussian Processes (GPs) provide superior performance in quantifying the risks of three uncertainty labels based on the negative log predictive probability (NLPP) evaluation metric and mean maximum predicted confidence levels (MMPCL), whilst retaining strong predictive performance.

**Discussion:**

Our conclusions highlight the utility of probabilistic models applied to “noisy” labels and that similar methods could provide utility for Natural Language Processing (NLP) based automated labeling tasks.

## 1 Introduction

Whilst current machine learning and deep learning models have shown great success in a variety of automated classification tasks (Rajkomar et al., 2018; Yang et al., 2018), sensitive healthcare contexts necessitate that these models quantify the risks of different diagnosis decisions, that is, *positive*—the illness is highly likely, *uncertain*—the illness is likely, with lower risks, *negative*—the illness is highly unlikely. The three uncertainty labels (also our class labels) allow each diagnosis decision to be made, by factoring in the degrees of uncertainty.

This is especially relevant as human annotation is very costly and model training data are often labeled by automatic labellers that use ontologies or are rule-based. For example, the UMLS (Unified Medical Language System) ontology (Bodenreider, 2004) is almost predominantly used to match linguistics patterns in clinical text to medical concepts [e.g., using the MetaMap tool (Aronson, 2006)]. The corpora annotated in this fashion are used to learn neural detectors of medical concepts in the self-supervised setting (Zhang et al., 2021). Rule-based automatic labellers such as CheXpert (Irvin et al., 2019), which builds on NegBio (Peng et al., 2018), use rules predefined by experts to extract clinical observations from the free text of radiology reports. Both use different rules, where CheXpert is more conservative, with more uncertain labels.

Most clinical Natural Language Processing (NLP) models focus on optimizing predictive classification performance on class labels with point estimates but pay less attention to further quantify the risks and uncertainty of these labels. Bayesian methods offer as a solution, a principled approach to produce well-calibrated uncertainty estimates. Popular Bayesian approximations (e.g., model ensembles and dropout techniques) have primarily been studied in the general NLP domain (Ovadia et al., 2019) or for continuous healthcare data (Combalia et al., 2020; Leibig et al., 2017). More rarely, those studies are in Clinical Natural Language Processing (NLP) (Yang et al., 2019; Guo et al., 2021; Popat and Ive, 2023), which has a unique asymmetric risk where underestimates and overestimates of confidence should be evaluated differently (e.g., pessimistic predictions may be needed for cases of early detection of rare conditions).

In this study, we build a model using Gaussian Processes (GPs) (Williams and Rasmussen, 2006), a Bayesian non-parametric method to quantify the uncertainty and disagreements associated with automatic data labels (non-parametric models investigate how the data relate rather than imposing of some type of relationship). Instead of learning distributions over model parameters as in Bayesian Deep Learning (BDL), GP models are non-parametric and learn distributions over functions (how probable are relationships between inputs and outputs), thus allowing for better generalization. The uncertainty estimates in a GP model come from its covariance function (or kernel) which determines how data points influence each other. GPs use this matrix to leverage the relationships between known data points and the new points to be predicted. It predicts the mean (expected values) and variance (uncertainty) of the posterior distribution for the new inputs.

We measure the quality of these uncertainty estimates based on the negative log predictive probability (NLPP) evaluation metric. It is directly related to the entropy (measure of uncertainty) of the predicted probability distribution. Lower negative log predictive probability indicates lower entropy, that is, more confident predictions.

We compare GP performance to two types of Bayesian approximations: frequentist ensembles using both Random Forests (RFs) (Breiman, 2001) and Deep Ensembles (ENS) (Lakshminarayanan et al., 2017), which are considered state of the art for uncertainty estimation in predictive settings (Lobacheva et al., 2020; Seedat and Kanan, 2019). We make use of the three automatic uncertainty labels (considered noisy labels), as provided by the CheXpert and Negbio labellers for diagnostics using radiology reports and make the following contributions:

(1) We study the quality of uncertainty estimates of a new range of uncertainty-aware models, previously understudied in clinical NLP;

(2) We successfully apply Sparse Gaussian Processes (GPs) to the diagnosis detection task in radiology reports. To the best of our knowledge, this is the first application in clinical NLP, and we demonstrate that GPs provide superior uncertainty representations yet retain comparable predictive performance when compared to baselines Random Forests and Deep Ensembles. We release the code with our experiments^1^;

(3) We demonstrate the utility of uncertainty estimates for the models trained with data labeled automatically (self-supervised) with disagreements (differences of opinions from rule-based CheXpert and Negbio labellers). GPs outperform the RF and ENS models, providing less certainty in the predictions for the test cases where labels assigned by different labellers disagree. Correct identification of such cases is crucial for reliable and trustworthy risk management, where any uncertain cases are referred for screening or to a human expert.

## 2 Methodology

Gaussian Processes (GPs) offer a principled probabilistic modeling approach and provide uncertainty estimates without needing post-processing, hence motivating our modeling decision. The GP is defined by GP(μ(**x**), **K**(**x**, **x**′, θ)), where **x** and **x**′ are input and training vectors, θ are the parameters of the covariance function, a mean function, and covariance function, respectively. When the GP is realized on observed data, it is given as $p\left(\text{y}|\text{f}\right)=\prod_{i=1}^{n}p\left({\text{y}}_{i}|{\text{f}}_{i}\right)$, where **f** is a vector of latent functions $p\left(\text{f}|\text{x},\theta \right)=\prod_{i=1}^{n}N\left({\text{f}}_{i};\text{0},{\text{K}}_{i}\right)$, which describe possible relations between training data and incoming samples. We refer the reader to Williams and Rasmussen (2006) for more details. In our study, we apply GPs to the classification task of clinical observation detection. The challenge with using GPs for classification is that an approximation for the posterior is required.^2^ GPs also have a complexity of *O*(*n*^3^) (Williams and Rasmussen, 2006), due to the matrix inversion, making it computationally intensive for NLP tasks using large datasets and high dimensional embeddings.

Hence, in dealing with the these challenges, we follow the standard domain practices and use Sparse GPs with Black-box variational inference (Dezfouli and Bonilla, 2015) (minimize the log-evidence lower bound) and use the Reparameterisation Trick (Kingma and Welling, 2022) to approximate the GPs. This is a more computationally feasible method, using a small set of latent inducing points. This approach is based on Titsias (2009). We also use automatic relevance determination (ARD) (MacKay, 1996) to allow for more flexibility in our kernel representation.

## 3 Experimental settings

### 3.1 Data

We study free-text radiology reports in the MIMIC-CXR database v2.0.0 (Johnson et al., 2019). The dataset is labeled by both the CheXpert (primary label) and Negbio labellers. CheXpert is based on Negbio, but they follow different strategies in mention detection: Negbio uses MetaMap, and CheXpert curates concepts predefined by clinical experts.

We analyse the “Oedema” pathology—65,833 reports with three uncertainty labels: *positive, uncertain*, and *negative* as explained earlier. Oedema was chosen due to the large data size, more inconsistent labels between the two labellers and more balanced split between classes. We partition the “Oedema” examples into (1) 63,482 consistent labels (~96% dataset), where both CheXpert and Negbio labels agree (CONS: 26,455—*positive*, 11,781—*uncertain*, 25,246—*negative*) and (2) 2,351 inconsistent labels making up ~4% of the total dataset, where CheXpert and Negbio labels disagree (INCONS, 522: *positive*, 1,317: *uncertain*, 512: *negative*). Validation and Test sets preserve the described class proportions and contain 10% of the dataset each.

In our setup, we use two variants of the inconsistent test sets (i.e., labellers disagree) to quantify the generalization to different labeling mechanisms namely, NegINCONSTest and CheXINCONSTest, taking as the “ground truth,” the automatic Negbio and CheXpert labels, respectively. CheXpert is considered the primary label; hence, the Train and Validation sets only use CheXpert labels, also containing both consistent and inconsistent data as mentioned above.

Note that so far whilst talking about uncertainty we have considered only predictive uncertainty, or uncertainty in the predictions, which can be decomposed into two types: aleatoric and epistemic (Hora, 1996; Hüllermeier and Waegeman, 2021). Aleatoric/data uncertainty in the output arises from incomplete information, noise, or class overlap in the dataset. We have eliminated it in the training data by considering only the CheXpert labels. We split the data into Train/Dev/Test two times and run our models on each split. The results shown in the study are averages of the two runs. Epistemic/model uncertainty is the uncertainty over which model parameters or functions best explain the observed data. We have addressed this uncertainty in the best way by choosing the GP model, which is a non-parametric model. The rest of the epistemic uncertainty lies with the hyperparameters chosen, such as the kernel, and this was minimized by trying different kernels at first.

Text pre-processing (tokenisation, lower-casing, white space, and punctuation removal) is done using Texthero. The average token length is ~43.5 tokens. Each training example's input data are represented by a 200 dimensional fixed length vector averaged over tokens. We use the biomedical Word2Vec-Pubmed word embeddings (Chiu et al., 2016) to represent tokens.

### 3.2 Models

Due to constraints in computational resources, our Sparse GP model consists of 300 inducing points and a radial basis function (RBF) kernel making use of ARD to learn the length scales. The expected likelihood term in *L*_*elbo*_ is estimated using Monte Carlo sampling. Following the best practices in the domain (Gal and Turner, 2015), the GP model is trained stochastically with RMSProp (learning rate = 0.003, epochs = 2, batch size = 500).

The RF model [using Scikit-learn (Pedregosa et al., 2011)] has the following hyperparameters based on random search over standard parameters: 300 trees and a max depth of 40 (the hyperparameters were chosen to match 300 inducing points of GP). Isotonic regression is used to obtain well-calibrated predictive distributions (again comparable to the GP).

The Deep Ensemble (ENS) model based on Lakshminarayanan et al. (2017) has five randomly initialized models [MLP with three-hidden layers with 200 hidden units/layer (with batch norm), Adam (learning rate = 3e-3), epochs = 10]. Following Lakshminarayanan et al. (2017), we use adversarial training [fast gradient sign method (Goodfellow et al., 2015)], for better model robustness.

### 3.3 Evaluation metrics

The models are evaluated based on predictive performance (accuracy), and we quantify the quality of the uncertainty representations using the negative log predictive probability *NLPP* = −*logp*(*C* = *y*_*n*_|*x*_*n*_). NLPP penalizes both overconfident predictions that are incorrect predictions and under-confident predictions that are correct. We optimize for higher predictive performance and lower NLPP.

Since we do not have ground truth labels for multiple human experts, we additionally assess the mean maximum predicted confidence level (MMPCL) of a certain test set, $MMPCL=\frac{1}{N}\overset{N}{\underset{n=1}{\sum }}max_{j}p\left(y_{n}=C_{j}|x_{n}\right)$, where *N* is total number of data points in the specific test set and *C*_*j*_ is per class label.

## 4 Results

We carry out two experiments: (1) We measure the performance in uncertainty representation and predictive performance of the model trained and validated on primary labels (CheXpert— CheXTrain and CheXVal, both contain consistent and inconsistent dataset) for the inconsistent Test data (disagreement- NegINCONSTest, CheXINCONSTest) as well as for the consistent Test data (agreement—CONSTest); (2) We conduct a group-wise performance analysis, particularly assessing how different models deal with the asymmetric risk of false negatives.

### 4.1 Performance across labeller agreement

#### 4.1.1 Inconsistent labels

Evaluating performance on inconsistent labels (CheXpert and Negbio disagree) highlights the use case of evaluation on out-of-distribution (OOD) labels (since the models are trained on CheXpert labels but tested on Negbio labels—NegINCONSTest). Table 1 illustrates the average predictive accuracy and average NLPP for all the test sets (NegINCONSTest, CheXINCONSTest, CONSTest). Overall, all models exhibit a higher NLPP across on both inconsistent test sets compared to CONSTest. The high NLPP highlights the models are able to correctly represent predictive uncertainty, when there was in fact disagreement in predictions.

**Table 1:** Results comparing GPs, RF, and ENS based on accuracy and NLPP for both inconsistent (OOD) and consistent test sets.

	NegINCONSTest		CheXINCONSTest		CONSTest	
	**Acc**↑	**NLPP**↓	**Acc**↑	**NLPP**↓	**Acc**↑	**NLPP**↓
**GP**	**0.294**	**1.234**	0.519	0.634	0.816	0.293
RF	0.271	1.451	**0.579**	**0.598**	0.795	0.316
ENS	0.263	1.281	0.468	0.691	**0.840**	**0.269**

Bold highlights the best results.

For experiments, comparing NegINCONSTest and CheXINCONSTest, we note that CheXpert has more conservative labels compared to Negbio: that is, CheXINCONSTest contains 63% of uncertain labels and 13% negative, whilst NegINCONSTest 30% uncertain and 63% negative. When the GP is evaluated on NegINCONSTest (labels are OOD) it outperforms the RF, yet the RF outperforms the GP on CheXINCONSTest (labels are in-distribution to training labels—CheXpert). In the case of ENS, it outperforms GP when evaluated on CONSTest but performs worse on inconsistent test sets (both CheXINCONSTest and NegINCONSTest). These results highlight the RF can be biased/overfit the “conservative” distribution of the training data, whereas the GP provides more “neutral” predictions, generalizing better to OOD data (e.g., NegINCONSTest). The poor performance of ENS on inconsistent test data [CheXINCONSTest (in-distribution), NegINCONSTest (OOD)], highlights that ENS does not generalize to OOD data nor does it handle noisy labels well. The GP generalization performance is highly desirable in medical settings, and our results on GP generalization are corroborated by Schulam and Saria (2017). For further analysis based on calibration, see Appendix.

#### 4.1.2 Consistent labels

As a control experiment and to account for the imbalanced data, we randomly sample the CONS test set (CONSTest) such that it has the same size as the other two inconsistent test sets. All models show lower NLPP compared to the inconsistent experiments as the cases within CONSTest tend to have high certainty/agreement.

### 4.2 Group-wise analysis for asymmetric risk

A significant asymmetric risk in the healthcare context lies in False Negatives (FN), that is, missing a positive diagnosis. Ideally, models should represent FNs with low predictive confidence, whilst True Positives (TPs) should have high predictive confidence. In Table 2, we compare the MMPCLs of FNs and TPs for the *positive* and *uncertain* labels for NegINCONSTest and CONSTest and ignore the *negative* labels.

**Table 2:** MMPCL results for GP, RF, and ENS for FNs and TPs evaluated on NegINCONSTest and CONSTest.

		NegINCONSTest		CONSTest	
		**Positive**	**Uncertain**	**Positive**	**Uncertain**
FN	GP	0.565^*^	**0.800**	0.647	**0.638**
	RF	**0.408** ^*^	0.857	0.657	0.661
	ENS	0.694	0.876	**0.624**	0.640
TP	GP	**0.711**	**0.605**	**0.797**	0.752
	RF	0.680	–	0.774	0.730
	ENS	0.670	0.318	0.780	**0.834**

Bold highlights the best results: lower confidence FN and higher confidence TP are desirable. RF did not produce any TP for Uncertain NegINCONSTest. ^*^Denotes that we have only one FN data point with positive label for this classifier.

On CONSTest (labellers agree), the GP provides lower MMPCL for FN predictions and higher MMPCL for TP predictions when compared to the RF across all test sets. Whilst ENS has good performance for FN positive and TP uncertain, this is traded off for reduced performance for FN uncertain and TP positive. We note that since GP has better balanced performance across both classes (positive and uncertain), it is likely to generalize better/not overfit, thus dealing with asymmetric risk in a more appropriate and balanced manner.

Similar patterns are observed for NegINCONS, with the GP better representing the asymmetric risk (for both positive and uncertain) when compared to both ENS and RF. However, the results are less conclusive due to the sparsity of FNs: we have only one FN data point with *positive* label for GP and RF (denoted by the *). Furthermore, the two data points are different; hence, the result where the RF outperforms on this subset cannot be considered as reliably representative. That said, the absence of TP uncertain labels for RF model mirrors the disagreements between the two labellers and highlights the inherent difficulty in predicting uncertain cases.

Overall, the GP and ENS give similar performance for in-distribution test data CONSTest; however, on the OOD test data NegINCONSTest, the GP gives better performance.

## 5 Conclusion

Current computational models in support of clinical decision-making are mostly unaware of uncertainty. In the modern healthcare systems, patients are often treated according to preset treatment pathways. This can improve outcomes as long as the correct diagnoses are made initially. However, there is often some degree of uncertainty in initial assessments, and this can be lost over the patient's journey. A prediction model that can detect and interpret this uncertainty could be used to highlight when alternative diagnoses and decisions need to be considered. This is an important step toward more flexible, interpretable, and trustworthy predictive models for clinical decision-making, which is an important goal in AI.

In this study, we have studied the performance of a Sparse GP model for clinical NLP that is capable of quantifying uncertainty in predictions. This model can be particularly advantageous in self-supervised learning scenarios with automatically labeled datasets (a very common scenario in Clinical NLP). It predicts lower confidence estimates for the test examples where different labeling heuristics tend to disagree, thus representing and carrying uncertainty throughout computational models to support clinical decision-making for more reliable, informed decisions.

In addition, overall our sparse GP provides more conservative predictions by showing lower confidence in incorrect predictions. This is important in healthcare contexts where potential harms from a model error can be very extensive as compared to singular errors of a doctor. This also implies that our model could be used to flag clinical notes for further review (e.g., by a human expert).

Whilst this study is focussed on uncertainty in labeling for medical tasks, we believe it highlights the utility of probabilistic models applied to “noisy” labels and that similar methods could provide utility for NLP based automated labeling tasks. Furthermore, we hope that this study will spur further research in this understudied area of automated labeling in clinical NLP—where annotated data are limited and expensive.

## 6 Limitations

Whilst our study presents an advancement in applying GPs to Natural Language Processing (NLP) problems, several limitations should be mentioned. We have organized them in the following sections below.

### 6.1 Modeling

#### 6.1.1 Hyperparameter tuning

Due to time and computational resource limitations, this project utilized only the RBF kernel, though other kernels such as Matern and convolutional kernels could potentially enhance performance, especially for high-dimensional data (van der Wilk et al., 2017). In addition, the GP model was run using only 300 inducing points. Exploring models with higher numbers of inducing points could yield better results. Future study should compare the GP model with state-of-the-art deep learning models, such as Transformer, to evaluate their effectiveness in handling data uncertainty.

#### 6.1.2 Decision boundary

For practical application in medical diagnosis, it is necessary to select a decision boundary based on prediction confidence of the model itself to filter out uncertain diagnoses effectively. This will allow the model to filter out those diagnoses that are truly uncertain and positive for manual revisions and also make sure the model has an optimal False Negative rate.

### 6.2 Data

Another limitation of this study is that only a single diagnosis Oedema with only less than 4% of the inconsistent data was explored. The labels used are not the ground truth. In the future, it would be helpful to evaluate the models against the ground truth labels and to get a better idea of the models' true performance. We suspect this performance will depend on how much humans will agree with the automatic annotation.

In general, GP models usually do not work well with large data sizes (like over 10K), and this study uses over 60K data. We hence believe the results could be further improved with sampling or cross-validation.

This study used fairly standard word embeddings, which are generally regarded as “show features”. More could be done to improve the data representation, such as using dependency graphs (Irvin et al., 2019), ontology-informed word embeddings (Zhang et al., 2021), or other relevant feature extraction tools. We believe those less shallow representations could again improve performance.

## Figures and Tables

**Figure A1:**
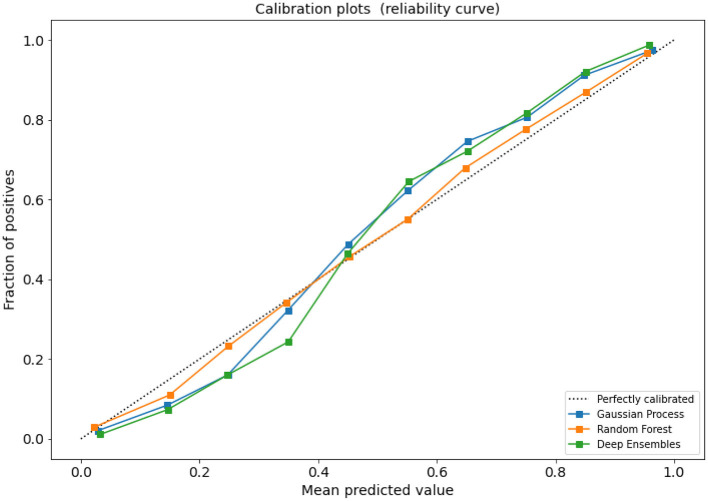
Reliability diagram for the CONSTest set: fraction of positives (*y*-axis) vs. mean predicted value (*x*-axis).

## Data Availability

Publicly available datasets were analyzed in this study. This data can be found at: https://mimic.mit.edu/docs/gettingstarted.

## Footnotes

^1^The code will be made available upon acceptance.

^2^Since the likelihood can no longer be assumed Gaussian due to the discrete labels, computing the posterior requires sampling as it becomes computationally intractable and hence, requires methods such as Markov Chain Monte Carlo and Variational Inference to approximate the posterior.

## Appendix

### Calibration

Figure A1 compares the calibration of the GP, RF, and ENS models, which all generate probabilities close to the optimal curve. Whilst, seemingly the calibrated RF model (using Isotonic Regression) gives more optimal predictions than the GP and ENS models, this result is based on the CONS test set (which is the same as the training distribution where both CheXpert and NegBio labels agree). However, both the GP and ENS are slightly less optimal. ENS is a neural model, and these models are known for poor calibration even when they exhibit very good performance (Guo et al., 2017) like in our case (see Table 1 where ENS shows the best performance for CONSTest). GP generalizes better across labellers, and hence, the GP overfits less to CONS specifically, when compared to RF.

### Examples of false negatives

A data point from consistent test set labeled *Positive* classified as *Negative*, (paraphrased)

Box 1 AGAIN MILD PROMINENCE OF THE PULMONARY INTERSTITIAL MARKINGS **SUGGESTIVE OF PULMONARY OEDEMA**, STABLE. SUBSEGMENTAL ATELECTASIS AT THE LUNG BASES. THERE IS NO DEFINITE CONSOLIDATION. NO PNEUMOTHORACES ARE SEEN.

A data point from inconsistent test set labeled *Positive* classified as *Negative*, (paraphrased)

Box 2 THE PATIENT IS STATUS POST DUAL-LEAD LEFT-SIDED AICD WITH LEADS EXTENDING TO THE EXPECTED POSITIONS OF THE RIGHT ATRIUM AND RIGHT VENTRICLE. PATIENT IS STATUS POST MEDIAN STERNOTOMY. THERE IS PROMINENCE OF THE CENTRAL PULMONARY VASCULATURE **SUGGESTING MILD OEDEMA**/VASCULAR CONGESTION. THE CARDIAC SILHOUETTE REMAINS QUITE ENLARGED. DIFFICULT TO EXCLUDE SMALL PLEURAL EFFUSIONS. THERE IS BIBASILAR ATELECTASIS. NO DISCRETE FOCAL CONSOLIDATION IS SEEN, ALTHOUGH OPACITY AT THE LUNG BASES PROJECTED ON THE LATERAL VIEW WHILE MAY BE DUE TO OVERLYING SOFT TISSUES, CONSOLIDATION IS DIFFICULT TO EXCLUDE.
